# Longitudinal association between an overall diet quality index and latent profiles of cardiovascular risk factors: results from a population based 13-year follow up cohort study

**DOI:** 10.1186/s12986-021-00560-5

**Published:** 2021-03-10

**Authors:** Fatemeh Nouri, Masoumeh Sadeghi, Noushin Mohammadifard, Hamidreza Roohafza, Awat Feizi, Nizal Sarrafzadegan

**Affiliations:** 1 Student Research Committee, School of Health, Isfahan University of Medical Sciences, Isfahan, Iran; 2 Department of Biostatistics and Epidemiology, School of Health, Isfahan University of Medical Sciences, Isfahan, Iran; 3 Isfahan Cardiovascular Research Center, Cardiovascular Research Institute, Isfahan University of Medical Sciences, Isfahan, Iran; 4 Cardiac Rehabilitation Research Center, Cardiovascular Research Institute, Isfahan University of Medical Sciences, Hezar Jerib Street, 8174673461 Isfahan, Iran; 5 Hypertension Research Center, Cardiovascular Research Institute, Isfahan University of Medical Sciences, Isfahan, Iran; 6 Heart Failure Research Center, Cardiovascular Research Institute, Isfahan University of Medical Sciences, Isfahan, Iran

**Keywords:** Cardiovascular disease, Risk factors, Overall diet quality, Latent variable model, Isfahan cohort study

## Abstract

**Background:**

Cardiovascular diseases (CVDs) are associated with an unhealthy lifestyle, including poor diet. Indices reflecting the overall quality of diets are more effective than single food or nutrient-based approaches in clarifying the diet disease relationship. The present study aims to use latent variable modeling to examine the longitudinal joint relationships between the latent profiles of CVDs risk factors and the diet quality index (DQI).

**Methods:**

A total of 4390 Iranian adults aged 35 and older within the framework of the Isfahan Cohort Study were included in the current secondary analysis. DQI focused on food groups, including fast foods, sweets, vegetables, fruits, fats, and proteins, based on a validated food frequency questionnaire. The score of DQI has a range between 0 (indicating healthy and high diet quality) and 2 (indicating unhealthy and low diet quality). Blood pressure (BP), anthropometric measurements, blood glucose, serum lipids, and high-sensitivity C-Reactive Protein (hs-CRP) were measured according to standard protocols in 2001, 2007, and 2013 to evaluate the profiles of CVDs risk factors. A Bayesian Multidimensional Graded Responses Linear Mixed Model was used for data analysis.

**Results:**

At baseline, the participants’ mean ± standard deviation age was 50.09 ± 11.21, and 49.5% of them were male. Three latent profiles of CVDs risk factors were derived: (1) Fit Pre-Metabolic Syndrome (FPMS) profile characterized by normal anthropometric indices and some impaired metabolic risk factors; (2) DysLipoproteinemia Central Obese (DLCO) profile with abdominal obesity and impaired low-density lipoprotein cholesterol as well as other normal risk factors; (3) Impaired Laboratory Inflammatory State (ILIS) profile with impaired high-density lipoprotein cholesterol and hs-CRP and other normal risk factors. In general, higher scores of the extracted latent profiles indicated more impaired function in the related risk factors. After controlling for various potential fixed and time-varying confounding variables, a significant positive longitudinal association was found between FPMS, DLCO, and ILIS profiles and DQI (β (95% CrI): 0.26 (0.03,0.51), 0.14 (0.01,0.27), and 0.24 (0.11,0.38), respectively), demonstrating that lower overall diet quality was associated with more impaired function of the related risk factors.

**Conclusions:**

More adherence to a healthy quality diet is associated with lower levels of all emerging latent profiles of CVDs risk factors. Increasing the knowledge of the community about the importance of the quality of consumed foods may help to prevent CVDs. It is recommended that further investigations, particularly interventional studies, be conducted to confirm our results.

**Supplementary Information:**

The online version contains supplementary material available at 10.1186/s12986-021-00560-5.

## Background

The Global Burden of Disease (GBD) study released that cardiovascular diseases (CVDs) as one of the most prominent non-communicable diseases (NCD) was the leading global cause of death since 1980 and a leading cause of disease burden in the Eastern Mediterranean Region [1]. CVDs are predisposed by various risk factors such as increased low-density lipoprotein cholesterol (LDL-C), decreased high-density lipoprotein cholesterol (HDL-C), high blood triglyceride concentrations (TG), high blood pressure (BP), hyperglycemia, high body mass index (BMI), abdominal obesity and high inflammatory state [1–4]. When the impairment coincides with risk factors, it leads to a dramatic increase in the incidence of CVDs and associated morbidity and mortality [5, 6].

The role of nutrition in the etiology of chronic diseases and its risk factors has been widely investigated. GBD 2017 declared that the dietary risks were responsible for 55% of CVDs deaths and 60% of disability-adjusted life years in low- and middle-income countries [7]. Several systematic reviews and meta-analyses of clinical trials and observational studies have indicated that adherence to a healthy diet is associated with decreased CVDs risk factors, incidence, and mortality [8–12]. However, there is inconsistency between an unhealthy diet and development of CVDs [7, 10, 13, 14].

Previous longitudinal studies assessing the effects of diet on CVDs and its risk factors were limited to specific food groups without considering synergic effects on health outcomes [15, 16]. Alternatively, they focused mainly on separately assessing risk factors without considering the interdependence of risk factors [17], or they were limited to particular groups of participants [12, 17], or considered the baseline assessment without considering the changes over time [18, 19]. In addition, most previous studies neglected the distinct combinations of risk factors and were limited to a simplistic approach with the adoption of ordinal or categorical unidimensional observed variables (i.e., metabolic syndrome, obesity/metabolic phenotype) [20, 21], leading to loss of information, and the failure to identify relationships among different variables affecting outcomes. Latent variable modeling can be used to define multi-component constructs like CVDs risk factors and to obtain useful information about the clustering of variables, along with controlling for measurement error. Measurement error can reduce the statistical power of study, yield biased or inconsistent parameter estimates, hide real effects and invert signs of the estimated coefficients [22, 23].

By focusing on tackling the above-mentioned challenges, the aim of the current study was to investigate the longitudinal joint relationship between the overall diet quality index (DQI) and the comprehensive composite assessments of CVD risk factors as latent profiles of CVD risk factors within a Bayesian Multidimensional Graded Responses Linear Mixed Model (MGRLMM) framework. The study was conducted in a representative and large sample of the general Iranian population who participated in the Isfahan Cohort Study (ICS). We extracted three comprehensive correlated latent variables as latent profiles of CVD risk factors, by combining repeated measurements of eight risk factors using the measurement part of the MGRLMM. In structural part of the MGRLMM, the latent profiles of CVD risk factors as multivariate dependent variables were regressed on the time-varying DQI as the independent variable controlling for the impact of fixed and time-varying confounding variables.

## Methods

### Study population

The current study was conducted under the framework of ICS. ICS is a community-based, longitudinal ongoing cohort study including a representative sample of 6504 adults aged 35 years old or over. The participants were selected from urban and rural areas of Arak, Isfahan, and Najafabad at baseline in 2001. Details about the multistage random sampling procedure (based on urban/rural, sex, and age distribution of the community), along with data collection and study design were published previously [24, 25]. The study was approved by the Ethics Committee of Isfahan Cardiovascular Research Center, a World Health Organization collaborating center. After obtaining informed written consent, physical examinations, laboratory measurements, and questionnaire-based interviews were performed in 2001 and repeated in 2007 and 2013. Of 6504 recruited participants in ICS in 2001, the subjects who had a previous history of CVDs, or those who were lost in follow-ups, or those who had missing data on the variables in the three phases of the study were excluded from the analysis. The reduced dataset consisted of 4390 subjects and 7121 observations from the participants during the study period, of which 2817, 2840, and 1464 observations were made in 2001, 2007, and 2013, respectively.

### Assessment of covariates

Trained health professionals conducted a detailed interview in 2001, 2007, and 2013 based on a questionnaire to identify the recruited participants’ sociodemographic characteristics, including age (years), sex (female/male), education years (0–5 year/6–12 years/ > 12 years), place of residence (rural/urban) and marital status (married/single, divorced, widowed)), as well as information on lifestyle behaviors, including physical activity, smoking status (ever smoke (current and past) /never smoke), and coping strategies. In addition, trained nurses and physicians performed physical examinations and medical interviews to determine the self and family history of hyperlipidemia, hypertension, diabetes, myocardial infarction, and stroke, as well as anti-dyslipidemia, hypertensive and diabetic medications used by the participants in three phases of the study.

Data on physical activity, expressed as metabolic equivalent task minutes per day (MET-m/d), were obtained through the International Physical Activity Questionnaire (IPAQ), whose reliability and validity were reported in previous studies [26, 27]. The whole score of physical activity was assessed based on four fields: homework, leisure time, worksite, and transportation. For the current analysis, we grouped total physical activity based on tertiles in each phase.

A validated two-factor 30-item stress management questionnaire was used to assess adaptive (20 items) and maladaptive (10 items) cognitive and behavioral coping strategies [28, 29]. Each item determines the frequency of using each strategy through a 3-point scale ranging from 1 (never), 2 (sometimes), and 3 (often). For scoring in each individual, the number of questions answered “often” is divided by the number of questions answered “often” and “sometimes” and is considered the percentage of maladaptive and adaptive skills. For the current analysis, we grouped maladaptive and adaptive coping strategies based on tertile in each phase.

### Assessment of CVDs risk factors

In this study, anthropometric measurements, serum concentrations of lipids, Fasting Blood Glucose (FBG), high-sensitivity C-Reactive Protein (hs-CRP), Systolic BP (SBP) and Diastolic BP (DBP) were measured according to standard protocols [25]. In the analysis, we considered following three categories for each CVDs risk factor (Normal, Borderline, Impaired), which was recommended by the Third Report of the National Cholesterol Education Program’s Adult Treatment Panel (ATP-III): BP (SBP)/DBP) ≤ 120/80 mm Hg, 120/80 to 140/90 mm Hg, and >  = 140/90 mm Hg; BMI (weight in kg divided by the square of height in meters) < 25 kg/m2, 25 to 30 kg/m2, and ≥ 30 kg/m2; LDL-C < 130 mg/dl, 130 to 160 mg/dl, and ≥ 160 mg/dl; TG < 150 mg/dl, 150 to 200 mg/dl, and ≥ 200 mg/dl; FBG < 110 mg/dl, 110 to 126 mg/dl, and ≥ 126 mg/dl; HDL-C > 60 mg/dl, 40 to 60 mg/dl, and < 40 mg/dl; [30]; hs-CRP < 1 mg/l, 1 to 3 mg/l, and > 3 mg/l; [30, 31] and waist circumference (WC) < 80 cm, 80 to 88 cm, and > 88 in females and < 94 cm, 94 to 102 cm, and > 102 in males [30, 32, 33].

### Dietary assessment

Dietary intake information was assessed by trained technicians, with a qualitative, 48-item food frequency questionnaire (FFQ) in the three phases of the study. A validation study comparing FFQ to the quantitative amount of food intake assessed by the mean of single 24-h recall and two food records revealed good relative reproducibility and validity of FFQ [34]. For each item in FFQ, the participants were asked about the frequency of consumption in the last preceding year on a daily, weekly, or monthly basis [34]. Data were converted to weekly consumption for analysis proposes. DQI focused on seven food groups, including fast food (4 items); vegetables and fruit (7 items); legumes, chicken, soy protein or fish (4 items); sweets (6 items); butter, hydrogenated oil, animal fats, or ghee (4 items); egg, whole dairy products, or meat (4 items); and olive and non-hydrogenated oil (2 items). Frequency responses in seven categories were scored as 2, 1, or 0, in which a higher score indicated a lower nutritional value. In calculating DQI, for example, for the ‘sweets’ category, a score of 2 was given for a frequency of four or more times weekly, a score of 1 was given to two or three times weekly, and a score of 0 was given to none or once per week. Details about the scoring method of DQI were published previously [35]. The whole score for DQI was considered by summing the answers to the responded items by each participant dividing by the number of items. The score of DQI has a range between 0 (indicating healthy and high overall diet quality) and 2 (indicating unhealthy and low overall diet quality).

### Statistical analysis

Data were analyzed using the R statistical software version 3.5.3 (R Core Team, 2019), and the Bayesian model fitting was performed in Stan (via RStan package, version 2.18.1) [36]. Moreover, using the Shinystan package (in R 3.5.3 environment), the Bayesian model convergence was monitored by Gelman-Rubin diagnostics (scale reduction R hat < 1.1) and trace plots (the absence of apparent trends).

Continuous and categorical data were summarized by the mean ± standard deviation (SD) and absolute frequencies (percentages), respectively. The linear mixed model, mixed effect logistic model, and the mixed effect ordered logistic model were performed to analyze continuous, dichotomous, and ordinal variables (Tables 1 and 2) across the three phases of the study, respectively.

**Table 1:** Characteristics of study participants in terms of lifestyle variables over phases of Isfahan Cohort Study (ICS)

Variables	Phases of study						Adjusted *P* value for trend
	2001		2007		2013		
Overall diet quality index (DQI)^1^	1.02 ± 0.26		0.74 ± 0.29		0.67 ± 0.28		< 0.001^5^
**Food groups (times per week)**^1^							
Non-hydrogenated and olive oil	2.28 ± 3.7		6.54 ± 4.63		6.35 ± 4.47		< 0.001^5^
Meat, egg and whole dairy products	6.38 ± 3.93		4.79 ± 3.15		2.04 ± 2.38		< 0.001^5^
Hydrogenated oil, ghee, animal fats and butter	10.19 ± 5.9		3.46 ± 4.26		1.95 ± 3.48		< 0.001^5^
Sweets	1.8 ± 3.23		1.3 ± 2.04		1.95 ± 3.18		0.14^5^
Legumes, chicken, soya protein and Fish	5.6 ± 3.11		5.72 ± 2.99		3.08 ± 2.48		< 0.001^5^
Vegetables and fruit	12.93 ± 6.71		13.17 ± 6.09		12.32 ± 6.39		0.002^5^
Fast foods	0.48 ± 0.96		0.28 ± 0.56		0.23 ± 0.53		< 0.001^5^
Family history^3^ (Yes) ^2^	1099(39.0)		1956(68.9)		1233(84.2)		< 0.001^6^
Anti-dyslipidemia medications (Yes)^2^	224(8.0)		441(15.5)		349(23.8)		< 0.001^6^
Anti-hypertensive medications (Yes) ^2^	320(11.4)		604(21.3)		393(26.8)		< 0.001^6^
Anti-diabetic medications (Yes) ^2^	112(4.0)		305(10.7)		224(15.3)		< 0.001^6^
Smoking status (ever smoke) ^2^	617(21.9)		489(17.2)		285(19.5)		< 0.001^6^
**Physical activity**^1^ **(MET-m/d)**^4^							
Tertile 1	< 565.7	332.01 ± 150.41	< 467.14	274.77 ± 122.74	< 501.55	296.36 ± 140.81	< 0.001^7^
Tertile 2	565.7–1062.8	810.97 ± 141.06	467.14–845.71	649.14 ± 107.51	501.55–856.14	670.37 ± 101.27	
Tertile 3	> 1062.8	1497.75 ± 435.75	> 845.71	1297.24 ± 491.34	> 856.14	1372.03 ± 582.31	
**Adaptive copping strategy**^1^							
Tertile 1	< 28.57	14.12 ± 7.52	< 20	8.03 ± 6.79	< 33.33	16.26 ± 11.03	0.61^7^
Tertile 2	28.57–69.23	48.33 ± 12.35	20–62.5	40.28 ± 12.10	33.33–75	53.89 ± 11.12	
Tertile 3	> 69.23	88.63 ± 9.76	> 62.5	84.42 ± 12.32	> 75	93.46 ± 8.56	
**Maladaptive copping strategy**^1^							
Tertile 1	0	0	< 14.29	0.03 ± 0.57	< 16.67	0	0.14^7^
Tertile 2	1–66.67	41.65 ± 10.84	14.29–66.67	41.06 ± 11.31	16.67–75	51.15 ± 15.00	
Tertile 3	> 66.67	90.87 ± 13.87	> 66.67	90.29 ± 13.88	> 75	99.23 ± 3.79	

^1^Data are expressed as Mean ± SD

^2^Data are expressed as number (percent)

^3^Family history of hyperlipidemia, hypertension, diabetes, myocardial infarction, and stroke

^4^MET-m/d: metabolic equivalent task minutes per day

^5^*P* value obtained from linear mixed model adjusted for sex and age

^6^*P* value obtained from mixed effect logistic regression model adjusted for sex and age

^7^*P* value obtained from mixed effect ordered logistic regression model adjusted for sex and age

**Table 2:** Cardiovascular Diseases (CVDs) risk factors of study population in three phases of Isfahan cohort study

CVDs risk factors	Assessed variables	Levels	Phases of study			Adjusted *P* value for trend
			2001	2007	2013	
Plasma glucose	FBG	Mean ± SD	87.74 ± 31.1	99.73 ± 35.85	107.1 ± 38.95	< 0.001^1^
		Normal (< 110)	2570 (91.2%)	2345 (82.6%)	1101 (75.2%)	< 0.001^2^
		Borderline (110–126)	87 (3.1%)	169 (6.0%)	137 (9.4%)	
		Impaired (≥ 126)	160 (5.7%)	326 (11.5%)	226 (15.4%)	
Blood pressure	SBP/DBP	Mean ± SD	120.22/77.67 ± 20.54/11.42	123.28/76.73 ± 19.83/11.62	128.36/83.64 ± 17.59/10.31	< 0.001^1^
		Normal (≤ 120/80)	1744 (61.9%)	1546 (54.4%)	563 (38.5%)	< 0.001^2^
		Borderline (120/80–140/90)	453 (16.1%)	573 (20.2%)	362 (24.7%)	
		Impaired (≥ 140/90)	620 (22.0%)	721 (25.4%)	539 (36.8%)	
Serum lipids	TG	Mean ± SD	173.38 ± 78.8	172.97 ± 111.67	155.59 ± 89.83	< 0.001^1^
		Normal (< 150)	1238 (43.9%)	1469 (51.7%)	854 (58.3%)	< 0.001^2^
		Borderline (150–200)	690 (24.5%)	539 (19.0%)	328 (22.4%)	
		Impaired (≥ 200)	889 (31.6%)	832 (29.3%)	282 (19.3%)	
	LDL-C	Mean ± SD	130.36 ± 42.11	124.14 ± 30.1	111.07 ± 27.76	< 0.001^1^
		Normal (< 130)	1456 (51.7%)	1691 (59.5%)	1114 (76.1%)	< 0.001^2^
		Borderline (130–160)	720 (25.6%)	814 (28.7%)	288 (19.7%)	
		Impaired (≥ 160)	641 (22.8%)	335 (11.8%)	62 (4.2%)	
	HDL-C	Mean ± SD	46.71 ± 10.11	45.89 ± 11.16	43.62 ± 10.88	< 0.001^1^
		Normal (> 60)	288 (10.2%)	288 (10.1%)	75 (5.1%)	< 0.001^2^
		Borderline (40–60)	1775 (63.0%)	1692 (59.6%)	845 (57.7%)	
		Impaired (< 40)	754 (26.8%)	860 (30.3%)	544 (37.2%)	
Anthropometric indices	BMI	Mean ± SD	26.57 ± 4.65	27.41 ± 4.39	27.96 ± 4.7	< 0.001^1^
		Normal (< 25)	1114 (39.5%)	865 (30.5%)	400 (27.3%)	< 0.001^2^
		Borderline (25–30)	1119 (39.7%)	1226 (43.2%)	622 (42.5%)	
		Impaired (≥ 30)	584 (20.7%)	749 (26.4%)	442 (30.2%)	
	WC (female/male)	Mean ± SD	94.11 ± 12.23	94.25 ± 11.37	97.31 ± 11.09	< 0.001^1^
		Normal (< 80 / < 94)	975 (34.6%)	934 (32.9%)	333 (22.7%)	< 0.001^2^
		Borderline (80–88/94–102)	547 (19.4%)	590 (20.8%)	328 (22.4%)	
		Impaired (> 88 / > 102)	1295 (46.0%)	1316 (46.3%)	803 (54.8%)	
Laboratory inflammatory state	hs-CRP	Mean ± SD	3.28 ± 1.51	4.97 ± 3.78	3.12 ± 4.38	< 0.001^1^
		Normal (< 1)	115 (4.1%)	407 (14.3%)	735 (50.2%)	< 0.001^2^
		Borderline (1–3)	1357 (48.2%)	726 (25.6%)	336 (23.0%)	
		Impaired (> 3)	1345 (47.7%)	1707 (60.1%)	393 (26.8%)	
Number of impaired CVDs risk factors (Impaired level vs. Borderline and Normal levels)		0	290 (10.29)	250 (8.8)	173 (11.82)	< 0.001^2^
		1	811 (28.79)	774 (27.25)	439 (29.99)	
		2	992 (35.21)	927 (32.64)	443 (30.26)	
		3	535 (18.99)	628 (22.11)	292 (19.95)	
		4	169 (6)	225 (7.92)	105 (7.17)	
		5	20 (0.71)	36 (1.27)	12 (0.82)	

FBG: Fasting Blood Glucose; SBP/DBP: Systolic/ Diastolic Blood Pressure; TG: Triglycerides; LDL-C: Low-Density Lipoprotein cholesterol; HDL-C: High-Density Lipoprotein cholesterol; BMI: Body Mass Index; WC: Waist Circumference; hs-CRP: high sensitivity C-Reactive Protein

^1^*P* value obtained from linear mixed model adjusted for sex and age

^2^*P* value obtained from mixed effect ordered logistic regression model adjusted for sex and age

We first conducted an exploratory factor analysis to examine the unidimensional assumption of 8 CVD risk factors based on the baseline data. Second, in order to evaluate the dimensionality, we applied multivariate analysis to extract multiple latent variables of the 8 CVDs risk factors using the measurement part of the MGRLMM via the Bayesian inference framework. In MGRLMM, eight CVDs risk factors were considered ordinal variables with three response categories (as listed above). In order to evaluate the number of latent variables of 8 CVDs risk factors as latent profiles, four different exploratory MGRLMMs with two, three, four, and five latent variables were fitted [37]. In MGRLMM, latent variables were considered as continuous variables, so that higher scores of latent variables indicated more impaired function in the related risk factors. We compared four competing MGRLMMs using Watanabe-Akaike information criterion (WAIC) and leave-one-out (LOO) cross-validation via LOO package in R3.5.3 environment. A lower score of both indices reflects the better the fitted model [38, 39]. In general, a common method for model selection is to balance the goodness of fit indices with the principle of parsimony (the lower number of parameters) and conceptual grounds [40].

To assess the longitudinal association of CVD risk factors with DQI considering the impacts of potential confounders, the structural part of MGRLMM was used [37]. In the structural part of MGRLMM, latent profiles were regarded as multivariate response variables while DQI was considered an independent variable. The model was adjusted for confounding variables and subject-specific random effects. In this model, in addition to independent and confounding variables being considered time-varying, the CVD risk factors changes were monitored during 13 years of follow-up. Advantages of MGRLMM include easy handling of the unbalanced structure of the dataset due to mistimed measurements and controlling for measurement error [22, 23, 37].

Crude and adjusted coefficients (β) and 95% credible interval (CrI) were presented in five different model strategies. We adjusted for demographic variables, including gender, age, marital status, education level, and place of residence in the second model. We further controlled for lifestyle-related variables, including smoking status, physical activity, and adaptive and maladaptive coping strategies of stress in the third model. Additional adjustments were made for anti-dyslipidemia, anti-diabetic, and anti-hypertensive medications in the fourth model. In the final model, a further adjustment was made for a family history of diabetes, hypertension, hyperlipidemia, myocardial infarction, and stroke. The statistical significance was assessed by 95% CrI in the Bayesian inference.

## Results

The participants’ mean ± SD age was 50.09 ± 11.21 years at baseline, and 49.5% of them were male. The majority (92.2%) of the participants were married, and approximately 72% were from urban areas in 2001. More than two-third of the participants (70.3%) was illiterate or had primary education, while approximately 6.2% of the participants had college education.

### Comparisons between lifestyle variables and phases of the study

Table 1 presents the lifestyle variables over the three phases of the study. The mean of DQI (as ranged from 0 to 2) was 1.02 ± 0.26, 0.74 ± 0.29, and 0.67 ± 0.28 in the observations of three phases of the study, respectively (adjusted P for trend < 0.001). The means of some food groups, including unhealthy fats (characterized by hydrogenated oil, ghee, animal fats, and butter), fast foods, and meat, egg, and whole dairy products, were considerably decreased during the study period (adjusted P for trend < 0.001). On the contrary, the result of the mixed effect model controlling for age and sex exhibited an increasing trend for healthy fats (characterized by non-hydrogenated and olive oil) from 2001 to 2013 (adjusted P for trend < 0.001). Besides, with regard to the trend analysis across follow-up time, family history and medications used were found to have an increasing trend over three phases of the study.

### Comparisons between CVDs risk factors and phases of the study

Table 2 presents the mean ± SD of CVDs risk factors and absolute frequency (percentage) across the three response categories of CVDs risk factors for the study participants in the three phases of the study. In addition, Table 2 displays the frequency of participants with different numbers of impaired CVDs risk factors. Results of mixed effect models adjusted for age and sex showed significant changes for all studied CVDs risk factors from 2001 to 2013. The mean BMI, FBG, WC, and BP were significantly increased during the study period (adjusted P for trend < 0.001). The mean values of all lipid variables exhibited a significantly decreasing trend from 2001 to 2013 (adjusted P for trend < 0.001). However, after an initial increase in 2007, hs-CRP was decreased in 2013 (mean ± SD in 2001: 3.28 ± 1.51, 2007: 4.97 ± 3.78, and 2013: 3.12 ± 4.38; adjusted P for trend < 0.001). Similar features were observed in terms of the participants’ frequency distribution across response categories of CVDs risk factors during the study period.

### Extraction of latent profiles of CVDs risk factors

According to the Scree plot of EFA, three latent factors based on eigenvalues (> 1) were identified (see Additional file 1: Fig. S1). Therefore, given this evidence, the unidimensional assumption is unreasonable in analyzing CVD risk factors.

Additional file 1: Table S1 (in Additional file 1) presents the factor loadings (95% CrI) of the different competing exploratory MGRLMMs for extracting the dimensions of latent profiles of CVDs risk factors. For identifiability during the model fitting, the loadings of some CVDs risk factors were restricted to 1. Moreover, the results of the goodness of fit indices for the four different MGRLMMs compared are presented in Additional file 1: Table S1 (in Additional file 1). In our data, the model with three latent variables was considered as the best model compared to other competing models due to better fit indices (the lower scores of LOO and WAIC), more parsimonious estimation (the lower number of parameters), and more interpretable latent variables.

Table 3 depicts how latent profiles of the best model were extracted from eight CVD risk factors using factor loadings based on the measurement part of MGRLMM. The first latent profile, namely Fit Pre-Metabolic Syndrome (FPMS) was characterized by normal anthropometric indices (BMI and WC) with some impaired risk factors (HDL-C, FBG, hs-CRP), but it did not meet the criteria to diagnose metabolic syndrome. The second latent profile, labeled as DysLipoproteinemia Central Obese (DLCO), contained abdominal obesity with impaired LDL-C as well as normal FBG, HDL-C, and hs-CRP. The third one, named Impaired Laboratory Inflammatory State (ILIS), was considerably loaded with impaired HDL-C and hs-CRP as well as normal LDL-C, FBG, WC, and BP. As Table 3 shows, for identifiability during the model fitting, the loadings of BMI, TG, and BP were restricted to 1 on first, second, and third latent profile, respectively. In general, higher scores of latent profiles indicate more impaired function in the related risk factors.

**Table 3:** Factor loading (95% credible interval) for CVDs risk factors in three extracted latent profiles of CVDs risk factors in Isfahan Cohort Study

CVDs risk factors	FPMS profile	DLCO profile	ILIS profile
BMI	1	0	0
TG	0	1	0
BP	0	0	1
LDL-C	0.06 (0.04, 0.08)	− 0.19 (− 0.26, − 0.13)	0.22 (0.16, 0.29)
FBG	− 0.10 (− 0.14, − 0.06)	0.66 (0.48, 0.85)	0.98 (0.92, 0.99)
HDL-C	− 0.11 (− 0.14, − 0.09)	0.99 (0.97, 0.99)	− 0.32 (− 0.39, − 0.23)
WC	0.98 (0.93, 0.99)	− 0.99 (− 0.99, − 0.96)	0.91 (0.77, 0.99)
hs-CRP	− 0.03 (− 0.05, − 0.01)	0.17 (0.11, 0.23)	− 0.09 (− 0.15, − 0.03)

FPMS: Fit Pre Metabolic Syndrome; DLCO: DisLipoproteinemia Central Obese; ILIS: Impaired Laboratory Inflammatory State; BMI: Body Mass Index; TG: Triglycerides; BP: Blood Pressure; LDL-C: Low-Density Lipoprotein cholesterol; FBG: Fasting Blood Glucose; HDL-C: High-Density Lipoprotein cholesterol; WC: Waist Circumference; CRP: high sensitivity C-Reactive Protein

### The longitudinal relationship between Diet Quality Index and latent profiles of CVDs risk factors

Table 4 represents the crude and adjusted models of the longitudinal joint associations of DQI with latent profiles of CVD risk factors based on the structural part of MGRLMM. Unadjusted and adjusted coefficients (β) and 95% CrI were presented in five different modeling strategies. In the crude model, we reached a significant positive joint association of each extracted FPMS, DLCO, and ILIS profile with DQI (β (95% CrI): 0.59 (0.36, 0.84), 0.35(0.20, 0.51), and 0.56(0.38, 0.79), respectively). In other words, higher scores of DQI (lower overall diet quality) were associated with higher scores of the latent profiles (more impaired function of the related risk factors), as anticipated. After controlling for demographic variables, higher scores of DQI were positively associated with higher scores of three profiles. The longitudinal association in later models was slightly reduced by further adjustment for other confounders, but this association remained significant. In the full adjusted model, a one-unit increase in the long-term DQI was associated with a 0.26-unit increase (β, 95% CrI: (0.03, 0.51)) in the score of the FPMS profile (more impaired HDL-C, FBG, and hs-CRP), a 0.14-unit increase (β, 95% CrI: (0.01, 0.27)) in the score of the DLCO profile (more impaired LDL-C and WC) and a 0.24-unit increase (β, 95% CrI: (0.11, 0.38)) in the score of the ILIS profile (more impaired hs-CRP and HDL-C).

**Table 4:** Bayesian estimated regression coefficients and 95% credible interval of the association of CVDs risk factors profiles and overall diet quality index in the Isfahan Cohort Study

		latent profiles of CVDs risk factors								
		FPMS profile			DLCO profile			ILIS profile		
		Coefficient	SD	95% credible interval	Coefficient	SD	95% credible interval	Coefficient	SD	95% credible interval
Diet quality index (DQI)	Model 1	0.59	0.12	(0.36, 0.84)	0.35	0.08	(0.20, 0.51)	0.56	0.11	(0.38, 0.79)
	Model 2	0.56	0.12	(0.32, 0.86)	0.41	0.07	(0.28, 0.54)	0.56	0.07	(0.42, 0.71)
	Model 3	0.54	0.12	(0.31, 0.78)	0.41	0.07	(0.27, 0.54)	0.57	0.07	(0.42, 0.71)
	Model 4	0.38	0.12	(0.14, 0.64)	0.17	0.06	(0.04, 0.29)	0.28	0.07	(0.15, 0.41)
	Model 5	0.26	0.12	(0.03, 0.51)	0.14	0.07	(0.01, 0.27)	0.24	0.07	(0.11, 0.38)

FPMS: Fit Pre Metabolic Syndrome; DLCO: DisLipoproteinemia Central Obese; ILIS: Impaired Laboratory Inflammatory State;

Model 1: unadjusted model

Model 2: adjusted for demographic variables (age, sex, education level, marital status and residency)

Model 3: adjusted for demographic variables and lifestyle-related variables (smoking status, physical activity and coping strategies)

Model 4: adjusted for demographic variables, lifestyle-related variables, and used medications (anti-dyslipidemia, anti-hypertensive, anti-diabetic medications)

Model 5: adjusted for demographic variables, lifestyle-related variables, used medications and family history of hyperlipidemia, hypertension, diabetes, myocardial infarction, and stroke

## Discussion

This study clarified the joint longitudinal association between an DQI and the latent profiles of CVDs risk factors among the Iranian population from 2001 to 2013. In this study, of 8 CVDs risk factors, three major profiles, FPMS, DLCO, and ILIS, were identified. The FPMS profile was characterized by normal anthropometric indices with some impaired risk factors, but it did not meet the criteria to diagnose metabolic syndrome. The DLCO profile contained abdominal obesity with impaired LDL as well as other normal risk factors. The ILIS profile was considerably loaded with impaired HDL and hs-CRP and other normal risk factors. After controlling for various likely fixed and time-varying confounding variables, DQI was significantly and positively associated with all identified profiles, meaning that lower overall diet quality was associated with more impaired function of the related risk factors.

In some previous studies, metabolic phenotypes characterized by glucose, lipid profiles, BP, and inflammation were evaluated according to different categories of BMI, WC, body fat percentage or body size, ranging from the metabolically healthy (0 to 1 cardio-metabolic abnormality) and normal weight (MHNW) to metabolically unhealthy (2 or more cardio-metabolic abnormalities) and overweight or obese (MUHO phenotypes) [41] and their associations with diet quality indices have been assessed [21]. The results of a meta-analysis of cohort studies showed that all metabolically unhealthy phenotypes (MUHNW, MUHO) were associated with an increased incidence of CVDs. In addition, MHO subjects had a raised risk of CVDs [41]. Regardless of the simplistic approach with the adoption of unidimensional categorical observed variable in previous studies and the advanced approach with the adoption of multidimensional continuous latent variables in the current study, some of the identified metabolic/obesity phenotypes are partly similar to the latent profiles of CVDs risk factors in the present study. Metabolically unhealthy normal weight phenotype (MUHNW) found in previous studies is in accordance with our FPMS and ILIS patterns. Furthermore, metabolically healthy overweight or the obese phenotype (MHO) found in previous studies is similar to our DLCO pattern. Results of a cross-sectional study of Brazilian adults indicated those who were in the fourth quartile of the data-driven unhealthy dietary pattern, characterized by condiments, oils, juice, snacks, sweets, soda, alcoholic beverages, had an increased occurrence chance of the MHO phenotype, being consistent with our results. Moreover, the top quartiles of this pattern were associated with an increased occurrence chance of the MUHO phenotype. However, there were no significant associations of these patterns with the MUHNW phenotype [21], probably due to the reverse causality in cross-sectional studies. Moreover, this finding might be attributed to the used simplistic method of assessing CVDs risk factors without considering the correlation between outcomes.

Regardless of separately assessing without considering the interdependence of risk factors in previous studies, there were several similarities between our findings and diet quality indices assessed in previous studies on different populations. Results of a cross-sectional sample of the Irish population showed enhanced diet quality assessed by Dietary Approaches to Stop Hypertension (DASH) was associated with a more favorable lipoprotein profile, BMI, WC, waist to hip ratio, and CRP. The DASH diet focuses on the consumption of vegetables, fruits, beans, nuts, low-fat dairy, and whole grains, and limiting intake of sugar-sweetened beverages, red meat, sweets, saturated, and total fat [42], which are relatively similar to food items included in DQI. In addition, consistent with our findings, results of a meta-analysis of randomized controlled trials including a 1917 American population showed that DASH diet interventions led to significant reductions in SBP, DBP, LDL-C and total cholesterol [12]. Likewise, the synthesis of the information from 5 clinical studies in a systematic review and meta-analysis showed that adherence to the Nordic dietary pattern focused on consumption of fruits, vegetables, whole grains, legumes, rapeseed oil, fish, shellfish, seaweed, as well as low intake of salt, sugar-sweetened products, high-fat dairy, and meat resulted in a significant decrease in SBP, DBP, total and LDL cholesterol levels [43]. Furthermore, results of a review on observational studies suggested that the higher Healthy Eating Index (HEI) as a diet quality index (characterized by grains, fruits, vegetables, meat/beans, milk, cholesterol, sodium, total fat, saturated fat, and variety of food consumption) was associated with lower weight gain [44, 45]. Moreover, the main findings of a meta-analysis including 1,020,642 subjects of cohort studies as well as its updated version, suggested that high-quality diets based on HEI, alternate HEI (AHEI), and DASH score, were associated with a significant decrease of risk in CVDs incidence and mortality [11, 46].

Several studies have investigated the association between data-derived dietary patterns identified by posteriori or hybrid methods and CVDs risk factors.

An observational study of 7646 healthy Italian adults by models accounting for demographic and lifestyle variables indicated that unhealthy dietary patterns (characterized by high intake of tomato sauce, red meat, pasta, alcohol, animal fats, eggs, processed meat, margarine, butter, sugar, and sweets) were associated with higher levels of FBG, serum lipids, CRP, BP, and CVDs risk score. However, a prudent pattern, assessed by high intake of fish, legumes, vegetables, soups, fruits, and olive oil was associated with lower levels [47].

In addition, results of a cross-sectional study in Yazd, central Iran, demonstrated that a higher score of a healthy dietary pattern (high intake of fruits, vegetables, tomatoes, yogurt drinks, and organ meats) was associated with lower levels of high-sensitivity CRP [48]. In another study on a 2,037 severely obese Swedish population, an identified unhealthy dietary pattern characterized by high intake of cheese, cake, chocolate, low-fiber bread, and fast food, and restricted intake of vegetables and fruit was associated with significantly higher SBP, DBP, WC, BMI, total cholesterol, and TG during 10 years of follow-up [17].

In general, the associations found between overall diet indices (assessed by diet quality indices or dietary patterns) and CVDs risk factors in previous studies were concordant with our results and those expected by the foods included in an overall diet. However, in the mentioned studies, an overall diet has been associated with CVDs risk factors without considering the correlation between the outcomes with a focus on separately assessing risk factors. It is now well known that clusters of interconnected risk factors produce CVDs [49]. Therefore, an effective way to deal with CVDs would be to regard multiple interconnected risk factors as clusters while considering the correlation between outcomes and distinguishing differences between distinct combinations of risk factors.

The potential mechanisms explaining the more impaired profiles of CVD risk factors with lower overall diet quality are multifactorial owing to the emphasis of DQI on the combinations of various food items and groups. Based on nutritional studies, these diet scores can provide synergic effects on health outcomes compared to the effects described for individual dietary components or nutrients [11, 50, 51]. Higher scores of the DQI result in higher intake of saturated and trans fat [35]. The previous evidence suggested an association between trans fatty acid intake and impaired lipid profiles as well as abdominal obesity [52–54]. Furthermore, higher scores of DQI indicate more consumption of sweet products and simple carbohydrates from refined sources. It is now well known that high refined carbohydrate diets are associated with CVDs risk factors, including impaired lipid profiles and obesity [55]. Moreover, higher scores on DQI show lower consumption of fiber and antioxidant sources, including fruits, vegetables, and legumes. The evidence indicates that these foods have anti-inflammatory effects, thereby improving the cardiovascular health [16, 56–58].

### Strengths and limitations

To the best of our knowledge, no longitudinal study with repeated measurements across time has investigated the association between overall diet quality and latent profiles of CVDs risk factors using an MGRLMM. Advantages of the model include easy handling of the unbalanced structure of the dataset due to mistimed measurements and controlling for measurement error. The model used in the analysis due to subject-specific random effects is an applied tool to consider a correlation and comorbidity between outcomes and effects of unobservable confounding factors. Additionally, a large and representative sample in this study enables us to have adequate power to interpret our results. In this study, in addition to DQI and confounding variables being considered time-varying, the CVD risk factors as multivariate response variables were monitored during a 13-year follow-up. However, the findings in the current study have some limitations. The FFQ used in the current study did not provide information on nutrient density and portion sizes; therefore, in the current study, we could not assess the participants’ whole energy intake. Furthermore, the self-reported diet may be systematically biased toward the under-outlining of energy-dense food groups and the over-outlining of healthy foods, particularly by abnormal-weight participants. Although we did not include some food groups in the calculation of DQI, future researchers may benefit from taking more comprehensive dietary indices into account. Moreover, it will be important to confirm our reported results in other populations considering regionally and culturally appropriate dietary patterns due to preserving cultural variety in health promotion [59]. We did not exclude a small number of participants who were underweight in the current study. However, the DQI of underweight participants had no significant difference compared with others. Dietary behaviors are likely to be affected by some characteristics of individuals. It is suggested that future studies considered the interaction effects of DQI and characteristics of individuals on latent profiles of CVDs risk factors. Repeated measurements of dietary intake and CVDs risk factors were all performed at the same time in our observational cohort study, which may lead to reverse causation bias. The clinical trial is thus needed to eliminate causality bias.

## Conclusions

This study supports the hypothesis of the joint longitudinal association between overall diet quality and comprehensive composite assessments of CVDs risk factors with considering the interdependence of risk factors. We found that more adherence to a healthy quality diet, characterized by high intakes of vegetables, fruits, legumes, chicken, soy protein, fish, and olive and non-hydrogenated oils, as well as low intakes of fast foods, sweets, butter, hydrogenated oil, animal fats, ghee, egg, whole dairy products, and meat, was associated with lower levels of CVDs risk factors. Increasing the knowledge of the community on the importance of a high-quality diet rich in important nutrients as well as implementing a well-planned community-based interventional program on lifestyle and risk factors may help in the prevention of CVDs in the country. Further investigations, particularly interventional studies, are needed to confirm our results considering total energy intake adjustment as well as prevention of other limitations in our study.

## Supplementary Information

**Additional file 1: Figure S1.** Scree plot of Exploratory Factor Analysis (EFA) for CVDs risk factors. Supplementary Table 1. Factor loading (95% credible interval (CrI)) for CVDs risk factors and model fit indices for all four competing Bayesian Multidimensional Graded Responses Linear Mixed Model (MGRLMM).

## Data Availability

The data sets used and/or analyzed during the current study are available from the corresponding author on reasonable request.

## Abbreviations

GBD: Global Burden of Disease
CVDs: Cardiovascular diseases
NCD: Non-communicable diseases
LDL-C: Low-density lipoprotein cholesterol
HDL-C: High-density lipoprotein cholesterol
TG: Triglycerides
BP: Blood pressure
BMI: Body mass index
DQI: Diet quality index
MGRLMM: Multidimensional Graded Responses Linear Mixed Model
ICS: Isfahan Cohort Study
MET-m/d: Metabolic equivalent task minutes per day
IPAQ: International Physical Activity Questionnaire
FBG: Fasting blood glucose
hs-CRP: High-sensitivity C-reactive protein
SBP/DBP: Systolic/diastolic blood pressure
ATP-III: Third Report of the National Cholesterol Education Program’s Adult Treatment Panel
WC: Waist circumference
FFQ: Food frequency questionnaire
SD: Standard deviation
CrI: Credible interval
FPMS: Fit Pre-Metabolic Syndrome
DLCO: DysLipoproteinemia Central Obese
ILIS: Impaired Laboratory Inflammatory State
MHNW: Metabolic healthy normal weight
MUHO: Metabolically unhealthy overweight or obese
MUHNW: Metabolic unhealthy with the normal weight
MHO: Metabolic healthy with overweight or obese
DASH: Dietary approaches to stop hypertension
HEI: Healthy eating index
AHEI: Alternate healthy eating index
