# Supplementary material for: Longitudinal association between an overall diet quality index and latent profiles of cardiovascular risk factors: results from a population based 13-year follow up cohort study
Source: Nutr Metab (Lond). 2021 Mar 10;18:28. doi: 10.1186/s12986-021-00560-5 (PMC7948330; doi:10.1186/s12986-021-00560-5)
Supplement: Supplementary file 1 — Additional file 1: Figure S1. Scree plot of Exploratory Factor Analysis (EFA) for CVDs risk factors. Supplementary Table 1. Factor loading (95% credible interval (CrI)) for CVDs risk factors and model fit indices for all four competing Bayesian Multidimensional Graded Responses Linear Mixed Model (MGRLMM). [file 12986_2021_560_MOESM1_ESM.docx]

**Supplementary Figure 1.** Scree plot of Exploratory Factor Analysis (EFA) for CVDs risk factors


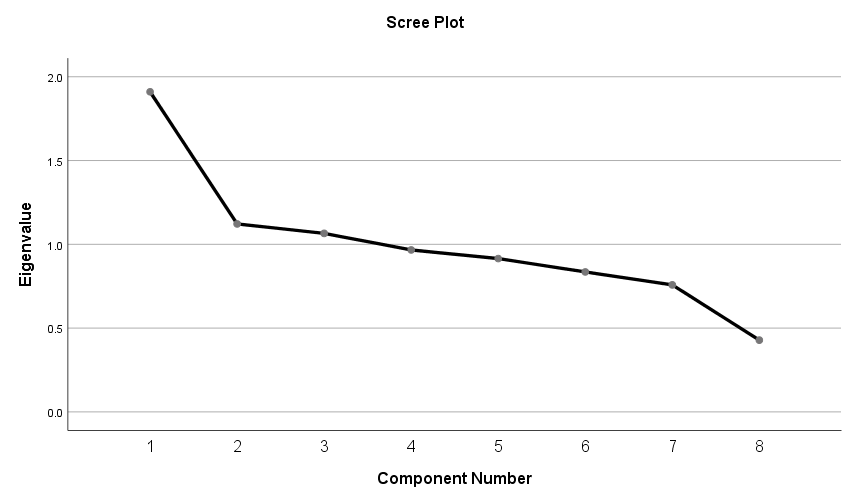


**Supplementary Table 1**. Factor loadings (95% credible interval (CrI)) for CVDs risk factors and model fit indices for all four competing Bayesian Multidimensional Graded Responses Linear Mixed Model (MGRLMM)

| CVDs risk factors | Two-Dimensional GRLMM | | Three-Dimensional GRLMM | | | Four-Dimensional GRLMM | | | | Five-Dimensional GRLMM | | | | | |
| --- | --- | --- | --- | --- | --- | --- | --- | --- | --- | --- | --- | --- | --- | --- | --- |
|  | Latent variable 1 | Latent variable 2 | Latent variable 1 | Latent variable 2 | Latent variable 3 | Latent variable 1 | Latent variable 2 | Latent variable 3 | Latent variable 4 | Latent variable 1 | Latent variable 2 | Latent variable 3 | Latent variable 4 | Latent variable 5 |  |
| BMI | 1 | 0 | 1 | 0 | 0 | 1 | 0 | 0 | 0 | 1 | 0 | 0 | 0 | 0 |  |
| TG | 0 | 1 | 0 | 1 | 0 | 0 | 1 | 0 | 0 | 0 | 1 | 0 | 0 | 0 |  |
| BP | 0.1  (0.08,0.13) | 0.19  (0.13,0.25) | 0 | 0 | 1 | 0 | 0 | 1 | 0 | 0 | 0 | 1 | 0 | 0 |  |
| LDL-C | 0.06  (0.04,0.08) | 0.04  (-0.02,0.11) | 0.06  (0.04,0.08) | -0.19  (-0.26, -0.13) | 0.22  (0.16,0.29) | 0 | 0 | 0 | 1 | 0 | 0 | 0 | 1 | 0 |  |
| FBG | 0.06  (0.03,0.10) | 0.47  (0.37,0.58) | -0.10  (-0.14.-0.06) | 0.66  (0.48,0.85) | 0.98  (0.92,0.99) | -0.04  (-0.08, -0.01) | 0.25  (0.15,0.34) | 0.97  (0.88,1.0) | 0.08  (-0.04,0.19) | 0 | 0 | 0 | 0 | 1 |  |
| HDL-C | -0.18  (-0.22, -0.14) | 0.86  (0.69,0.99) | -0.11  (-0.14, -0.09) | 0.99  (0.97,0.99) | -0.32  (-0.39, -0.23) | -0.09  (-0.12, -0.05) | 0.96  (0.88,1.0) | -0.18  (-0.31, -0.05) | -0.96(-1.0, -0.88) | -0.09  (-0.13, -0.06) | 0.96  (-.88,1.0) | -0.05  (-0.15.0.04 | -0.96  (-1.0, -0.86) | -0.04  (-0.09,0.01) |  |
| WC | 0.92  (0.83,0.99) | -0.23  (-0.33, -0.13) | 0.98  (0.93,0.99) | -0.99  (-0.99, -0.96) | 0.91  (0.77,0.99) | 0.73  (0.63,0.82) | -0.23  (-0.32, -0.16) | 0.16  (0.01, 0.31) | 0.53  (0.41,0.67) | 0.74  (0.66,0.83) | -0.26  (-0.36, -0.18) | -0.03  (-0.16,0.08) | 0.51  (0.38,0.63) | 0.10  (0.04,0.16) |  |
| hs-CRP | -0.05  (-0.07, -0.03) | 0.14  (0.09,0.19) | -0.03  (-0.05, -0.01) | 0.17  (0.11,0.23) | -0.09  (-0.15, -0.03) | 0.01  (-0.01,0.03) | 0.20  (0.15,0.26) | -0.37  (-0.47, -0.27) | -0.07  (-0.13, -0.001) | -0.01  (-0.03,0.01) | 0.20  (0.15,0.25) | -0.16  (-0.25, -0.08) | -0.07  (-0.12, -0.003) | -0.04  (-0.07, -0.01) |  |
| LOO (SE) | 95572.4 (298.0) | | 94298.7 (300.6) | | | 92997.2 (304.5) | | | | 91989.6 (305.0) | | | | |  |
| P_LOO_ (SE) | 5538.8 (41.5) | | 7292.6 (45.6) | | | 9172.6 (49.9) | | | | 10893.9 (60.6) | | | | |  |
| WAIC (SE) | 95078.2 (296.8) | | 93605.6 (298.9) | | | 92112.2 (302.3) | | | | 90512.3 (298.9) | | | | |  |
| P_WAIC_ (SE) | 5291.7 (39.4) | | 6946.1 (42.8) | | | 8730.1 (47.1) | | | | 10155.2 (55.2) | | | | |  |

CVD: Cardiovascular Disease; BMI: Body Mass Index; TG: Triglycerides; BP: Blood Pressure; LDL-C: Low-Density Lipoprotein cholesterol; FBG: Fasting Blood Glucose; HDL-C: High-Density Lipoprotein cholesterol; WC: Waist Circumference; CRP: high sensitivity C-Reactive Protein; LOO: leave-one-out cross-validation; SE: Standard Error; P: Estimated effective number of parameters; WAIC: Watanabe-Akaike information criterion; GRLMM: Graded Responses Linear Mixed Model.
